# Protective cardiovascular benefits of exercise training as measured by circulating endothelial cells and high-density lipoprotein in adults

**DOI:** 10.1016/j.jtumed.2021.12.003

**Published:** 2022-01-23

**Authors:** Kumboyono Kumboyono, Indah N. Chomsy, Dylan H. Firdaus, Meddy Setiawan, Titin A. Wihastuti

**Affiliations:** a School of Nursing, Faculty of Health Sciences, University of Brawijaya, Malang, Indonesia; b Doctoral Program of Medical Science, Faculty of Medicine, University of Brawijaya, Malang, Indonesia; c Master Program of Biomedical Science, Faculty of Medicine, University of Brawijaya, Malang, Indonesia; d Internal Medicine Department, Faculty of Medicine, University of Muhammadiyah Malang, Malang, Indonesia

**Keywords:** التمارين الهوائية, أمراض القلب والأوعية الدموية, حماية القلب والأوعية الدموية, الخلايا البطانية المنتشرة, كوليسترول البروتين الدهني عالي الكثافة., Aerobic exercise training, Cardiovascular disease, Cardiovascular protection, Circulating endothelial cells, High-density lipoprotein-cholesterol

## Abstract

**Objective:**

This study aims to determine the protective cardiovascular effect of aerobic exercise training by measuring cluster of differentiation 146 (CD146), circulating endothelial cell (CEC), and high-density lipoprotein-cholesterol (HDL-C) levels in adults.

**Methods:**

This study was an experimental pre-post-test without a control group. Forty-five participants were divided into three groups based on aerobic exercise training intensity: low, moderate, and high. Whole blood samples were measured for HDL-C levels. In addition, CEC was isolated from Peripheral Blood Mononuclear Cells (PBMC) samples, then identified by CD146 marker using flow cytometry.

**Results:**

CEC percentage and HDL-C increase after aerobic exercise training. There was a significant difference in CEC percentage between the intensity groups. However, there was no difference in HDL-C levels.

**Conclusion:**

Aerobic exercise training can protect cardiovascular health by stimulating CEC mobilization, identified by CD146. In addition, an HDL-C level increase also contributes to cardiovascular protection by decreasing inflammation levels, inhibiting low-density lipoprotein-cholesterol oxidation, improving endothelial regeneration capabilities, and lowering blood glucose.

## الملخص

**أهداف البحث:**

تهدف هذه الدراسة إلى تحديد التأثير الوقائي للقلب والأوعية الدموية للتمارين الهوائية المقاسة بواسطة الخلايا البطانية المنتشرة ذات مجموعة التمايز +146وكوليسترول البروتين الدهني عالي الكثافة في البالغين.

**طرق البحث:**

هذه الدراسة عبارة عن اختبار مسبق تجريبي بدون مجموعة ضابطة. تم تقسيم 45 مشاركا إلى ثلاث مجموعات بناء على كثافة التدريب الهوائي: كثافة منخفضة ومتوسطة وعالية. تم قياس عينات الدم من أجل مجموعة التمايز +146 للخلايا البطانية المنتشرة باستخدام قياس التدفق الخلوي ومستويات كوليسترول البروتين الدهني عالي الكثافة.

**النتائج:**

أظهرت نتائج هذه الدراسة زيادة ذات دلالة إحصائية للخلايا البطانية المنتشرة وكوليسترول البروتين الدهني عالي الكثافة بعد التمرين. كان هناك فرق كبير بين مجموعة الشدة في نسبة الخلايا البطانية المنتشرة، ولكن لم يتم العثور على أي منها في مستويات كوليسترول البروتين الدهني عالي الكثافة.

**الاستنتاجات:**

يمكن أن يحمي التدريب الهوائي صحة القلب والأوعية الدموية عن طريق زيادة تكيف الأوعية الدموية عن طريق زيادة الخلايا البطانية المنتشرة ذات مجموعة التمايز +146. تساهم زيادة مستوى كوليسترول البروتين الدهني عالي الكثافة أيضا في حماية القلب والأوعية الدموية عن طريق تقليل مستويات الالتهاب وتثبيط أكسدة كوليسترول البروتين الدهني قليل الكثافة وزيادة القدرة على تجديد البطانة وتقليل نسبة الجلوكوز في الدم.

## Introduction

Cardiovascular disease (CVD) is a non-communicable disease involving the heart and blood vessels, including coronary artery disease, stroke, arrhythmia, and other heart diseases.^1^ According to the World Health Organization, CVD is estimated to cause 17.9 million deaths every year worldwide.^2^ It is the leading cause of death, covering approximately 31% of deaths globally.^3^^,^^4^ Many factors can cause CVD, including obesity, smoking, diabetes mellitus, unhealthy diet, lack of physical activity, inflammation, and dyslipidaemia.^2^^,^^5^ Despite decreased exposure to some risk factors, exposure to other risk factors is still high, especially a lack of physical activity.^2^

Endothelial dysfunction (ED) plays an essential role in the pathogenesis, progression, and prognosis of CVD.^5^ ED is characterized by an imbalance between the release of vasodilators and an increase in pro-thrombotic, pro-inflammatory, and pro-constrictive factors.^6^ Together with inflammation and free radicals, ED causes atherosclerosis characterized by blood flow blockage.^6^ In addition, endothelial cells are sensitive towards oxidative stress, which often occurs in various CVD risk factors, including diabetes, dyslipidaemia, smoking, and hypertension.^5^ This pathological condition leads to structural damage in the vascular wall, causing endothelial cell detachment.^6^

Several mechanisms, including apoptosis, damaged endothelial structures due to cytokines, mechanical injury, and atherosclerosis, will cause the release of mature endothelial cells from blood vessels, called circulating endothelial cells (CEC).^7^ Although CECs are rarely found in the circulation of healthy individuals, populations with specific conditions, such as inflammation, infection, metabolic disease, and CVD, have elevated CEC levels.^8^ Several marker proteins can identify CECs, including cluster of differentiation (CD)31, CD62E, CD54, CD106, CD151, and CD146.^9^ The most commonly used method to identify CECs is flow cytometry CD146 as a marker protein.^10^ A previous study stated that CECs could identify vascular damage and correlate with disease activity.^5^^,^^9^ In addition to their role in vascular damage, CECs increase and migrate from healthy segments to repair the injured part of blood vessels and contribute to endothelial cell regeneration and angiogenesis.^11^

Cardiovascular health is also related to high-density lipoprotein (HDL) levels. HDLs are lipoproteins that bind to high-density cholesterols and have preventative CVD risk functions.^12^ This function is highly associated with reverse cholesterol transport (RCT), which transports excessive cholesterol in peripheral tissues to the liver.^13^ HDL consists of apolipoprotein A1 (ApoA-1), synthesized in the liver and gastrointestinal tract and secreted into the plasma.^12^ Previous research stated that HDLs in healthy individuals could protect the cardiovascular system through various mechanisms.^14^ For example, HDLs can increase endothelial nitric oxide synthase (eNOS) expression and stimulate nitric oxide (NO) release from endothelial cells, reduce white blood cell adhesion by decreasing the expression of vascular cell adhesion molecules, and enhance endothelial repair after vascular injury.^15^^,^^16^ Furthermore, HDLs can promote endothelial regeneration indirectly through endothelial progenitor cells by increasing NO synthesis.^17^

Several efforts can be made to maintain cardiovascular health, one of which is aerobic exercise training. When exercising, the body experiences a hypoxic condition caused by high physical activity and stimulates compensation from the cardiovascular system to induce anti-atherogenic adaptations in vascular structures.^18^ Other studies state that aerobic exercise training could improve vascular function via several mechanisms, including endothelial cell regeneration capabilities.^19^^,^^20^ Furthermore, aerobic exercise training is an easy and inexpensive method widely prescribed to prevent CVD.^21^ However, few studies explain the effect of aerobic exercise training and its intensity on CEC levels and its correlation with HDL levels serving as a biomarker of cardiovascular health.^6^ Thus, this study aimed to determine the molecular mechanism of aerobic exercise training in preventing CVD by observing CEC and HDL levels).

## Materials and Methods

### Experimental design

This study was designed as an experimental pre-post-test without a control group. Research intended to determine how aerobic exercise training and its intensity affect CEC percentage and NO expression.

### Study participants

The study population included 45 participants aged 19–30 years old divided randomly into three groups equally, based on the intensity of the aerobic exercise training: low-, moderate-, and high-intensity groups. Participants were healthy individuals without any history of metabolic syndromes, namely diabetes, obesity, or dyslipidaemia. Participants also did not have a history of chronic diseases, including malignancy, autoimmune diseases, and other chronic diseases. All the participants gave informed consent, expressing their willingness to complete the research procedure.

### Aerobic exercise training procedure

Aerobic exercise training involved running or jogging for 30 min, three times a week for eight weeks. The participant's heart rate determined the intensity of the exercise. The low-, moderate-, and high-intensities were targeted to reach 50–71%, 71–80%, and 81–90% of the maximum heart rate, respectively. If the participant did not reach the target within 30 min, they repeated the aerobic exercise training for 30 min and the heart rate measurement.

### HDL level and CEC percentage measurements

Complete blood count and lipid profile analyses were done at the Central Laboratory of Saiful Anwar Hospital Malang. CEC samples were obtained via the peripheral blood sampling process and separated to attain peripheral blood mononuclear cells (PBMCs). The CEC percentage was measured using flow cytometry at the Biology Laboratory of Brawijaya University, following the protocol provided by the manufacturer (BD FACSCalibur™, Becton, Dickinson and Company, Franklin Lakes, New Jersey, United States). First, CECs were identified using anti-human PE-conjugated CD146. Then, a 15 mL tube was prepared with ficoll in a ratio of 1:1 with the number of blood samples taken. The blood sample from the EDTA tube was slowly poured into the tube. The tube containing the sample was centrifuged at 2600 rpm for 30 min. The buffy coat from the centrifugation was pipetted and transferred to a 15 mL tube and supplemented with PBS to a volume of 10 mL. The tube was then re-centrifuged at 2600 rpm for 10 min. After the supernatant was removed, staining buffer and fixation buffer were added to the formed pellet. Then, 5 μL of CEC marker was added and incubated for 20 min in a dark room. Finally, the results were read using a flow cytometer.

### Statistical analysis

Statistical analysis was performed using the IBM® SPSS® version 23.0 (New York, United State). An ANOVA test was used to determine the difference between participant characteristics in each group. A Wilcoxon test was conducted to determine changes in the amount of CECs and HDLs after the aerobic exercise training treatment. A Kruskal–Wallis test was conducted to determine the difference in aerobic exercise training intensity between the groups regarding CEC and HDL levels (α = 0.05).

## Results

### Baseline characteristics of participants

Participant characteristics consisted of age, sex, weight, height, and body mass index (BMI) (Table 1). The majority of participants were women (73.3%). The division of participant groups based on aerobic exercise training intensity was homogeneous, and there was no significant sex difference between the groups (*p* = 0.376). The age of the participants ranged from 19 to 30 years, thus forming a group of young adults with no history of CVD, metabolic disease, or chronic diseases. There was also no significant difference in weight (*p* = 0.165) and BMI (*p* = 0.075) between the groups, meaning the division of the participant groups was homogeneous.

**Table 1:** Baseline characteristics based on the aerobic exercise training intensity group.

Variable	Exercise intensity			*p*-value
	Mild (n = 15)	Moderate (n = 15)	High (n = 15)	
**Age (yearsold)**				<0.001∗
$\bar{\text{x}}$ ± SD	22.8 ± 1.70	24.2 ± 1.50	25.0 ± 1.20	
Sex **(%)**				0.376
Male	3 (20.0)	3 (20.0)	6 (33.3)	
Female	12 (80.0)	12 (80.0)	9 (66.7)	
**Weight (kg)**	60.6 ± 10.5	52.8 ± 12.2	58.9 ± 12.1	0.165
**Height (cm)**	157 ± 2.1	157 ± 4.8	161 ± 5.5	0.024∗
**BMI (kg/m**^**2**^**)**	24.8 ± 4.6	21.3 ± 3.6	22.8 ± 4.0	0.075

Note: ∗Data were analysed using a one-way ANOVA test with a significance level of 5%.

**Figure 1:**
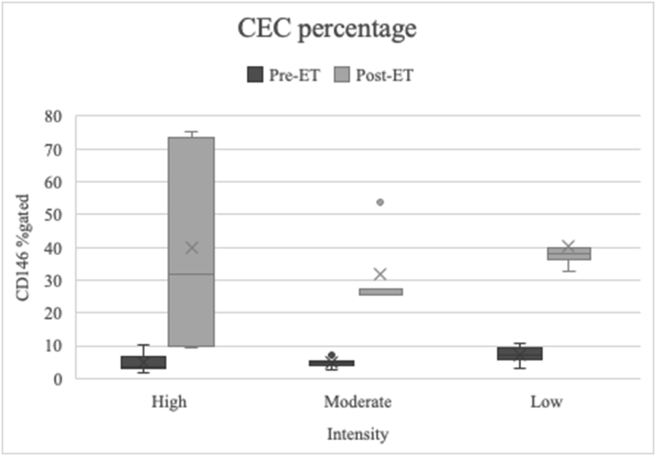
Percentage of CD146 CEC in blood samples. There was a significant increase in the percentage of CD146 CEC after the participants completed the aerobic exercise training (*p* < 0.001). Moreover, there was significant difference in the percentage of CD146 CEC between the aerobic exercise training intensity (*p* = 0.031).

**Figure 2:**
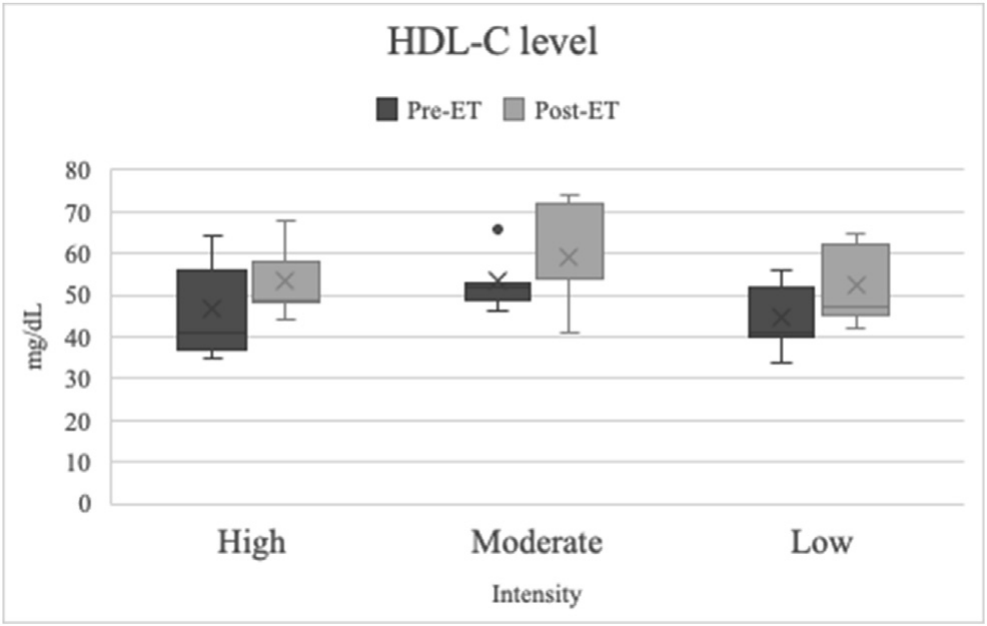
HDL-C levels (mg/dL) in blood samples. There was a significant increase in the HDL-C levels after the participants completed the aerobic exercise training (*p* < 0.001). However, there was no significant difference in the HDL-C levels between the aerobic exercise training intensity (*p* = 0.258).

### Effect of aerobic exercise training on CEC percentage

As shown in Table 2, there was a significant difference in CEC percentage in all the participant groups after being prescribed aerobic exercise training. The data analysis showed a significant increase in CEC percentage after being prescribed aerobic exercise training (*p* < 0.001). After being given aerobic exercise training, there was also a significant difference in CEC percentage between the intensities (*p* = 0.031) (Figure 1). The most significant change occurred in the low-intensity group compared to the moderate- and high-intensity training groups. A significant difference in HDL levels in all the participant groups was also be found (Figure 2). The data analysis showed that aerobic exercise training prescription caused a significant increase in HDL levels (*p* < 0.001). However, unlike the CEC percentage, there was no significant effect of different aerobic exercise training intensity prescriptions on HDL level (*p* = 0.258). Aerobic exercise training increased HDL levels by 17.0%, 9.83%, and 14.78% in the low-, moderate-, and high-intensity aerobic exercise training, respectively.

**Table 2:** Statistical analysis of the aerobic exercise training effect on CEC percentage and HDL levels in the blood.

Variable	Exercise intensity			*p*-value
	Mild (n = 15)	Moderate (n = 15)	High (n = 15)	
**CEC (CD146) (percent gated)**				
Pre-test	4.86 ± 2.8	4.86 ± 1.60	5.01 ± 3.17	0.057^a^
Post-test	40.5 ± 8.0	31.9 ± 11.4	39.9 ± 30.3	0.031^a^
Δ Post-Pre-test	33.3 ± 9.3	27.0 ± 12.6	34.9 ± 28.4	<0.001^b^
**HDL (mg/dL)**				
Pre-test	44.6 ± 8.42	53.2 ± 7.09	46.0 ± 11.8	0.062^a^
Post-test	52.2 ± 9.74	59.0 ± 12.8	53.4 ± 8.91	0.258^a^
Δ Post-Pre-test	7.60 ± 4.88	5.80 ± 9.54	6.80 ± 3.67	<0.001^b^

Note: All numbers presented in the table are mean ± SD.

a Data were analysed using Kruskal–Wallis test with a significance level of 5%.

b Data were analysed using Wilcoxon test with a significance level of 5%.

## Discussion

### Aerobic exercise training improved the CEC percentage

This study showed a significant increase in CEC percentage following aerobic exercise training prescription (*p* < 0.001). Aerobic exercise training induces several mechanisms that stimulate cardiovascular responses. Aerobic exercise training generates higher shear stress against the vessel wall due to increased blood flow. Shear stress is a mechanical force that can induce endothelial cell detachment from the vascular wall. However, the vascular wall can respond against shear stress, mainly through vasodilatation caused by mechanoreceptors, stimulating eNOS expression and NO release. Routine aerobic exercise training causes repeated haemodynamic stimulation, inducing anti-atherogenic adaptation in vascular structures.^18^ Angiogenesis is a vascular adaptation that increases blood flow following nutrition and oxygen demands caused by increased physical activity. Collateral blood vessels, especially in the myocardium, can maintain perfusion when coronary arteries are blocked to reduce myocardial infarction lesions.^22^ The process of formation of new blood vessels is followed by the proliferation and migration of endothelial and progenitor cells.

CD146, a standard protein marker for CECs, was used to identify CECs using flow cytometry. CD146 is a cell-surface molecule from the immunoglobulin superfamily, expressed in endothelial cells, including CECs.^23^ Under physiological conditions, CD146 expression is restricted in normal tissue and is an adhesion molecule. Increased CD146 expression allows cells to interact with other cells, promoting cell proliferation and migration, especially in actively proliferating cells.^24^ CD146 also plays a vital role in angiogenesis as a co-receptor of pro-angiogenic factor receptors, such as vascular endothelial growth factor (VEGF).^24^ CD146 is often used to identify CECs as a CVD indicator.^10^ In previous studies, CECs increased in a pathological condition where the endothelial injury occurred.^5^^,^^6^ However, another study stated that CECs also play a part in endothelial repair.^11^ To our knowledge, this is the first study that specifically uses CD146 to identify CECs in young adults prescribed aerobic exercise training as a method to observe the vascular response. Therefore, the usage of CD146 in an aerobic exercise training study is a novelty.

Previous studies stated that exercise induces angiogenesis to fulfil tissue demands, especially muscle capillary formation.^25^ Aerobic exercise training can cause hypoxia conditions, stimulate hypoxia-inducible factor-1α (HIF1A), and increase VEGF expression, acting as an angiogenesis factor.^7^^,^^26^^,^^27^ Angiogenesis requires the proliferation of endothelial cells and progenitor cells, including CD146 endothelial cells. Thus, increasing levels of CD146 CECs after aerobic exercise training indicates that physiological angiogenesis is an adaptation mechanism. This study shows that different degrees of vascular adaptation were also present. There was a significant difference in CEC percentages between the intensity groups (p = 0.031). The high-intensity group showed the highest CEC percentage increase, followed by low- and moderate-intensity aerobic exercise training, respectively.

### Aerobic exercise training improved HDL levels

This study showed a significant effect (*p* < 0.001) of aerobic exercise training prescription on increasing HDL-cholesterol (HDL-C) levels in young adults. The HDL structure largely consists of various particles of different sizes and apolipoproteins, such as ApoA-I and ApoA-II.^28^ HDL performs cardioprotective functions mainly through RCT by removing excess cholesterol from macrophages on the artery walls and transporting it back to the liver.^12^ The amounts of released cholesterol from peripheral cells represent the HDL-C levels.^14^ Various factors can affect blood cholesterol levels, including dietary and environmental factors.^29^ Studies suggest that dietary cholesterol increases serum total cholesterol and low-density lipoprotein-cholesterol (LDL-C) and HDL-C levels.^29^ Other factors contributing to CVD, including physical inactivity, obesity, smoking, and diabetes mellitus, are related to low HDL-C levels.^30^ These risk factors are essential in the pathogenesis of ED leading to atherosclerosis.^5^ Regular aerobic exercise training and a healthy diet are the primary interventions in managing blood lipid profiles, including HDL-C, and preventing CVD.^31^ Although many studies have shown that HDL-C is related to cardiovascular health, only a few have linked the effect of exercise on HDL-C levels.

Several studies have reported the changes in lipid profiles after aerobic and resistance aerobic exercise training.^31^^,^^32^ Previous studies documented increased HDL-C and decreased LDL-C after aerobic training.^32^ In contrast to LDL-C and total cholesterol, HDL-C represents the cardiovascular health status because it removes cholesterol from peripheral cells. Aerobic exercise training contributes to various mechanisms that prevent CVD. Some studies argue that aerobic exercise training could increase antioxidant expression, reduce inflammatory cytokines, induce NO synthesis, and increase endothelial regeneration capabilities.^18^^,^^26^^,^^33^ These functions are highly related to the ability of HDL-C particles to transport vitamins, hormones, and antioxidants.^32^ In addition, increased HDL-C levels contribute to vascular protection effects, such as decreased inflammation levels, LDL-C oxidation inhibition, blood glucose reduction, and various other beneficial vascular health effects.^13^^,^^14^^,^^32^ In this study, different aerobic exercise training intensities had no significant effects. However, the most notable increase in HDL-C level was observed in the low-intensity group. The molecular mechanism of aerobic exercise training to increase HDL-C levels needs to be evaluated further in future studies.

This study was limited to aerobic exercise and did not include resistance exercise. This was because this study aimed to observe the response of the cardiovascular system to aerobic exercise, which can reportedly stimulate anti-atherogenic adaptations through various mechanisms. Total and LDL-C were also not analysed in this study. Additional studies need to address these limitations and explore how aerobic exercise affects total and LDL-C.

## Conclusion

This study observed a significant increase in CEC percentage and HDL-C levels in the blood after prescription aerobic exercise training in adult humans. The CD146 CEC percentage increase is a form of vascular adaptation to fulfil oxygen and nutrition demands through angiogenesis. Different intensities of aerobic exercise training showed varying degrees of vascular adaptation. HDL-C crucially contributes to cardiovascular health protection via various mechanisms, including decreasing inflammation levels, inhibiting LDL-C oxidation, increasing endothelial regeneration capabilities, and reducing blood glucose. These effects have shown the importance of aerobic exercise training in protecting cardiovascular health and preventing CVD risk. Further research should combine these molecules with others to study the benefits of aerobic exercise training on cardiovascular health.

## Source of funding

The Ministry of Research, Technology, and Higher Education of the Republic of Indonesia through 10.13039/501100006374Universitas Brawijaya (Number 537.12.2/UN10.C10/PN/2021) funded the study materials and medical writing for this research.

## Conflict of interest

The authors have no conflicts of interest to declare.

## Ethical approval

The participants gave informed consent and signed to express their willingness to participate in this study. The study was granted ethical clearance by the ethics committee of the Faculty of Medicine, Brawijaya University (Letter Number 149/EC/KEPK/08/2020) on August 19, 2020. Furthermore, all of the patients who participated in this study provided informed consent per the Declaration of Helsinki.

## Authors contributions

KK conceived and designed the study. INC and DHF drafted, did the data entry, prepared the tables and figures, and edited the manuscript. KK and TAW collected data. KK, MS, and TAW revised the methods and performed the statistical analysis. TAW supervised all aspects of the study. All authors have critically reviewed and approved the final draft and are responsible for the content and similarity index of the manuscript. All authors have checked and approved the final approval of the manuscript.
